# Motor rehabilitation using virtual reality

**DOI:** 10.1186/1743-0003-1-10

**Published:** 2004-12-10

**Authors:** Heidi Sveistrup

**Affiliations:** 1 School of Rehabilitation Sciences, Faculty of Health Sciences, University of Ottawa, Canada

## Abstract

Virtual Reality (VR) provides a unique medium suited to the achievement of several requirements for effective rehabilitation intervention. Specifically, therapy can be provided within a functional, purposeful and motivating context. Many VR applications present opportunities for individuals to participate in experiences, which are engaging and rewarding. In addition to the value of the rehabilitation experience for the user, both therapists and users benefit from the ability to readily grade and document the therapeutic intervention using various systems. In VR, advanced technologies are used to produce simulated, interactive and multi-dimensional environments. Visual interfaces including desktop monitors and head-mounted displays (HMDs), haptic interfaces, and real-time motion tracking devices are used to create environments allowing users to interact with images and virtual objects in real-time through multiple sensory modalities. Opportunities for object manipulation and body movement through virtual space provide frameworks that, in varying degrees, are perceived as comparable to similar opportunities in the real world. This paper reviews current work on motor rehabilitation using virtual environments and virtual reality and where possible, compares outcomes with those achieved in real-world applications.

## Introduction

One of the major goals of rehabilitation is to make quantitative and qualitative improvements in daily activities in order to improve the quality of independent living. Three determinants of motor recovery are early intervention, task-oriented training, and repetition intensity [1] while a major objective of rehabilitation is to identify the means to provide repeated opportunities for tasks that involve multimodal processes (different sensory modalities including vision, haptics, proprioception, audition) and that further enable increases in function. Carr and Shepherd [2] focus on motor relearning where relearned movements are structured to be task specific. They suggest that the practice of specific motor skills leads to the ability to perform the task and that motor tasks should be practiced in the appropriate environments where sensory inputs modulate their performance. The functional relevance of the specific environmental context has been specifically addressed by Keshner and colleagues [3-5] as it relates to posture control. These authors have shown that specific postural responses differ between paradigms where isolated individual control pathways are manipulated (i.e., visual, vestibular, somatosensory pathway) as opposed to within a functionally relevant context where information from multiple pathways is available.

The successful integration of virtual reality into multiple aspects of medicine, psychology, and rehabilitation has demonstrated the potential for the technology to present opportunities to engage in behaviors in challenging but safe, ecologically valid environments while maintaining experimental control over stimulus delivery and measurement [for review see [6,7]]. Moreover, in VR, the user (patient, therapist) interacts with a multidimensional, multisensory computer generated environment, a virtual environment, which can be explored in real time [8]. Virtual reality also offers the capacity to individualize treatment needs while providing increased standardization of assessment and training protocols. In fact, preliminary evidence [9-11] indicates that VR provides a unique medium where therapy can be provided within a functional, purposeful and motivating context and can be readily graded and documented.

Several features distinguish virtual environments from other forms of visual imaging such as video and television. A key feature of all VR applications is interaction. Virtual environments (VE) are created that allow the user to interact with not only the VE but also with virtual objects within the environment. In some systems, the interaction may be achieved via a pointer operated by a mouse or joystick button. In other systems, a representation of the user's hand (or other body part) may be generated within the environment where movement of the virtual hand is "slaved" to the user's hand allowing a more natural interaction with objects. Finally, while many applications of VR allow the user to control the viewpoint on the screen, third-person views or images of the users themselves that appear as players in the environment also provide the opportunity for interaction with the VE.

A broad range of visual interfaces are used to create varying degrees of immersion in a VE ranging from conventional desktop monitors to head mounted displays. Increasingly complex, fully immersive VR systems, such as the Cave Automatic Virtual Environment (CAVE) developed at the University of Illinois at Chicago, provide the illusion of immersion by projecting stereo images on the walls and floor of a room-sized cube. Several persons wearing lightweight stereo glasses can enter and walk freely inside the CAVE. A head tracking system continuously adjusts the stereo projection to the current position of the leading viewer. In order to integrate the movement of the user with that of the VE and virtual objects, user position and motion must be tracked so that virtual images can be updated in real-time. Motion tracking approaches include color subtraction technology, video frame subtraction as well as magnetic and infrared tracking devices. Technical advances in the development of these interfaces have minimized the once lengthy lag times responsible for some of the earlier reports of cybersickness.

To date, rehabilitation applications have primarily used visual and auditory sensory input while the addition of haptics is less developed. Haptic interface devices including gloves, pens, joysticks and exoskeletons provide users with a sense of touch and allow the user to feel a variety of textures as well as changes in texture. There is increasing evidence that haptic information is an effective addition towards the accomplishment of certain treatment objectives such as increasing joint range of motion and force [12]. Haptic information has also been identified as a significant signal for improving a subject's performance in more difficult tasks. For example, Shing and colleagues [13] report a specific benefit of adding haptic information to an upper extremity movement when the difficulty of the task, in this case a 3D pick and place task, was high. Integration of visual and haptic interfaces with motion tracking allows the user to become immersed in three dimensional virtual environments, including three dimensional sound, and virtual objects that can be picked up, manipulated, and even felt with the fingers and hands [14].

Another cardinal feature of virtual reality is the provision of a sense of actual presence in, and control over, the simulated environment [15]. The sense of presence has been defined as the feeling of being in an environment even if one is not physically present and resulting in behavior that is congruent with the subject's situation in the environment [16]. Early studies relied on questionnaires to characterize presence within a virtual environment [15] with more recent work suggesting that physiological measures including heart rate and galvanic skin response provide important information about user immersion [17].

### Movement elicited and generated in virtual reality applications

One important consideration with the application of virtual reality and movement in virtual environments is the behavior or movement characteristics of subjects in virtual environments [8]. Recent work by Feldman and colleagues [18] specifically compared movements made with or to virtual objects in a VE to movements made with or to real objects in real environments. Virtual representations of the hand were obtained by combining a fiber optic glove with a prehension force feedback device. Orientation of the hand in the VE was achieved using an electromagnetic tracker while kinematic data of the arm and trunk were recorded as the participant reached separately to real and virtual targets. Minimal movement differences in spatial and temporal kinematics of reaching in healthy adults were identified and included the amount of terminal wrist and elbow extension as well as timing of maximal grip aperture. There were no differences in movement characteristics between the real and virtual task in participants with hemiparesis. The authors suggest that VR is similar enough to reality to provide an effective training environment for rehabilitation.

In contrast, we have demonstrated significant differences between functional lateral reach performances when performed in the real environment versus in a virtual environment delivered on a flatscreen [19]. The VR technology, VIVID Group's IREX system, provided participants with a third-person view of the users themselves in the virtual environments where they acted on virtual objects. Both young and old adults reached significantly further when virtual objects were presented in the VE compared to when reaches were made to real objects presented in the periphery. Lateral stability is crucial for performance of many weight-bearing tasks including turning, transferring, and stepping onto a stool while controlling a reach made as far as possible to the side requires regulation of the position of the center of mass within the limits of stability. We proposed that embedding the reaching task within a VR application may have resulted in shifting attention away from the potential for loss of balance, whereas focusing attention on balance, such as in the real-environment, may have resulted in increased fear of destabilization and underestimation of true ability.

Improving the functional abilities of patients is commonly achieved by using tasks of increasing difficulty in combination with physical and/or verbal guidance of the patient's movements or actions. Thus, integrating the means to modulate the level of difficulty within a VR task is of crucial importance. A virtual reality system (VIVID GX) was used to provide independent leisure opportunities to adults with cerebral palsy and severe intellectual disabilities who were non-speaking and who used wheelchairs for mobility [15]. The participants demonstrated an exceptional degree of enthusiasm during the VR experiences reacting with appropriate, goal-oriented responses. However, a small number of participants clearly displayed involuntary movement synergies, increased reflexes and maladaptive postures, which were attributed to the level of task difficulty. The ability to change the virtual environment relatively easily, to grade task difficulty and to adapt it according to the patient's capabilities are important advantages of VR, since these features are essential to cognitive and motor remediation [20].

### Does the technology work?

#### Transfer of training

Central to the issue of virtual environments as a training medium is the issue of transfer of training; does task improvement or learning transfer reliably from a VE to a real environment? Virtual environments and VR interventions should not only be used to augment current ability or to provide exposure to "other" therapeutic possibilities, but importantly to demonstrate distinct carryover to real-life functional tasks. One major challenge is identifying effective and motivating intervention tools that enable transfer of the skills and abilities achieved during rehabilitation to function in the "real" world. For example, recent studies stress that simple repetitive movements of an affected limb are not productive for the reorganization process but that it is action related to skill acquisition which contribute to the desired effect [21].

Rose and colleagues studied the transfer of training of a simple sensorimotor virtual task to performance on the "real world" equivalent [22]. The real-world equivalent consisted of a curved wire suspended between two vertical supports. With the non-preferred hand, the subject held a rod with a wire loop at the end and guided the loop along the wire without touching it. Contact between loop and wire, defined as an error, produced feedback. Errors and time to complete task were recorded. The group provided with no practice did significantly worse that the two practice groups, one practicing with the virtual task and one practicing with the real task, although with no difference between the type of practice performed. In other words, within the constraints of this task, final real-world performance benefited as much from real as virtual practice. Thus, it is not sufficient simply to demonstrate that training does transfer in a given situation. It is crucial to identify whether a specific skill or a general familiarity with the training context is being transferred. If specific skills are transferred, it is important to determine whether the transferred training lasts as long and as reliably as an equivalent amount of real world training [22]. In addition, the conditions such as degree of immersiveness, overlap between real and virtual tasks, must be understood if we are to optimize or facilitate transfer.

#### Balance and Posture

Several systems have been used in studies of balance including a combined HMD display system combined with a fixed bicycle, a flatscreen VR system providing primarily 2D visual information and more recently an immersive dynamic virtual environment combined with a posture platform.

Kim et al [23] reported preliminary data from healthy adults using a bicycle linked to a virtual visual environment and suggested that this training system would be beneficial for postural balance control. They described decreases in cycling path deviation and increases in cycling velocity following a short training period and suggested that these variables, in conjunction with additional parameters, may be relevant for determining a training effect on balance rehabilitation. Several problems remain to be resolved including the limited integration of bicycle motion and auditory cues. A specific concern is that a fixed bike was used which could provide the degree of safety necessary for an individual with a significant amount of balance impairment. However, a fixed bike sets up incongruence between the expectation of lean/tilt of the bike when covering a curved path and the sensory information indicating no tilt.

Multiple applications of flatscreen VR for balance training have been reported that have used video capture technology from VividGroup's GX or IREX systems [see for example, [9,10,24-26]]. The systems take a video image of the user and use color subtraction software to remove a monochrome background and insert the user into a virtual environment. Proprietary software is used to allow the user to interact with virtual objects within the VE. Applications that have been used in various studies include: 1) a juggling task where the participant is required to reach laterally to juggle virtual balls; 2) a conveyer belt task where the participant is required to turn sideways, pick up a virtual box from a virtual conveyer belt, turn and deposit the box on a second virtual conveyer belt; and 3) a snowboard task where the user is required to lean sideways to avoid trees, rocks and other virtual objects while boarding down a hill. The applications are modifiable allowing the task difficulty to be modified by increasing the number of virtual objects to contact, increasing the speed at which the objects or environment moves, or increasing and decreasing the height of the objects requiring users to reach to the ground or to step up onto a stool. One of the earliest reports of use of the technology in rehabilitation compared therapy delivered through VR to a conventional approach in a sample of frail, older adults [25]. Greater improvements in dynamic standing tolerance were reported for a small (n = 3 to 4) group of older adults following a VR therapy than for a small group (n = 3 to 4 per group) following a standard occupational therapy program.

We have used a similar approach with a significantly larger study population of community-living individuals with traumatic brain injury [see [9,10,26] for preliminary data]. A six week, three sessions per week intervention trial compared an activity-based exercise program (ABE) with a VR-based exercise program (VRE). Both exercise programs resulted in clinically significant changes on the Community Balance and Mobility Scale (CB&M) [27], used to measure functional mobility and balance, with average improvements of 6 and 10 points recorded for the ABE and VRE groups, respectively. Although not all participants involved in the exercise programs improved on their balance measures, 10 out of 14 individuals in the VRE group and 4 out of 10 individuals in the ABE group had clinically significant improvements. Most recently, we have demonstrated significant improvements in balance and functional mobility in community-living older adults following a VR exercise program. The comparison group completed a biofeedback exercise program and also demonstrated significant balance improvement [24]. Although these two studies did not demonstrate significantly greater improvements in balance outcome with the VR exercise program relative to the comparison intervention, other benefits of VR were identified. Specifically, the participants in the VR programs indicated greater enthusiasm about the exercise programs and reported greater enjoyment and improved confidence. The implications of these psychosocial benefits for long-term exercise compliance and participation have yet to be determined.

More recently, Keshner and colleagues [4] have united an immersive dynamic virtual environment projected onto a wall with a linear accelerator (sled) that is translated in the anterior-posterior direction. Study participants stand on the sled in front of a screen on which a virtual image is projected. Various combinations of inputs (i.e., translating the support surface, moving the virtual scene, or combining different motions) are used to determine responses elicited when conflicts of different magnitudes between visual and vestibular/somatosensory signals are delivered. The results of initial experiments clearly demonstrate the non-linear effect in the postural response from single versus different combinations of inputs. These findings suggest that using this or similar complex, multimodal environments for rehabilitation intervention would promote ongoing recalculation of sensory inputs that would result in appropriate updates of posture within realistic environmental contexts.

#### Locomotion

Patients with Parkinson's disease akinesia have little difficulty stepping over objects in their path even when they are totally unable to initiate a step on open ground [28]. A virtual display superimposed over a user's visual field, augmented reality, has been shown to initiate and sustain walking in akinetic Parkinson's patients. Reiss and colleagues [28] reported that a stable cue appearing about six inches in front of the toes was required to initiate the first step, while cues scrolling toward the feet, as if stable on the ground as the person moves, were needed to sustain walking. The effectiveness of the visual cue was dependent on the degree and type of akinesia with, as a general rule, more realistic cues needed as the severity of akinesia increases.

A locomotor interface, GaitMaster2 (GM2), intended to provide the user with the sense of forward movement while his/her actual position in space is constant, has been tested with two individuals with hemiplegia following a stroke [29]. The user stands on two footpads that move individually with each user's foot providing a sense of movement over a virtual terrain. The footpads in the GM2 follow the trajectory of a healthy individual when walking. The user thus experiences a corrected foot trajectory for each step. Modifications in gait patterns of two hemiplegic patients following gait training with the GM2 included moderate improvements in gait speed, improvements in leg muscle activity, increased symmetry during gait and improvement in QOL.

A VR-enhanced orthopedic appliance for use with individuals with spinal cord injuries has also been developed and links a gait-inducing exoskeleton to a HMD providing binocular visual displays [30]. Briefly, the exoskeleton consists of a semi-rigid sling that supports the bust and lower limbs of the user. The sling is equipped with small actuators that move the lower extremities in accordance with human gait. Preliminary results from two experimental sessions with the same patient, a 26-year old with complete paraplegia, showed improvements in self-confidence, higher levels of optimism and motivation as well as increased relaxation and activity scores.

A novel VR application for locomotor rehabilitation couples a three dimensional visual scene with a self-paced treadmill [31]. Briefly, both treadmill speed and scene progression are based on real-time feedback of subject position and progression with the speed of walking adjusted easily by the individual user. Preliminary trials of the system provided subjects with varying levels of interaction with the scene surface and surrounding objects with a strong sense of presence reported by users. Ongoing work by the group includes development and evaluation of a training protocol for locomotor rehabilitation in individuals with stroke.

#### Upper and Lower Extremity Function

Several upper and lower extremity VR applications have been developed using different technologies. Preliminary data suggest potential benefits of various systems. For example, a report based on two case studies using the Vivid GX video capture technology demonstrates improvements in upper extremity function [32]. The first individual had a T9 complete spinal cord injury requiring use of wheelchair for all mobility activities. His primary rehabilitation goal was to improve sitting balance in order to enable him to perform functional activities such as reaching out for a book placed on a shelf. Analysis of videotaped records of performance revealed that initially he used only one hand at a time to interact with the virtual objects while leaving the other on his lap or on the wheelchair arm rest in order to maintain balance. As sessions with the VR system progressed, he began to use both hands during the tasks relying on weak trunk muscles to maintain balance. The second individual had a right hemispheric stroke and ambulated with a cane due to poor control of foot and poor standing balance. He had functional movement in the upper extremity, suffered from mild attention deficit and required some help when dressing the lower extremity. The application he used consisted of balls appearing in the VE from all sides requiring that he pay attention to the entire visual space. After 3 minutes of interaction, he asked to get up and continue with therapy while in a standing position (although therapist behind was necessary for safety). Both participants reported enjoyment and wanted to repeat experience if possible. Importantly, they acknowledged the relevance of the experience to their rehabilitation process.

Holden and colleagues [33] developed a VE training system based on the principle of learning by imitation. Pre-recorded movements of a virtual 'teacher' are displayed as either movements of the limb's endpoint or as an entire arm. Patient movements are recorded using an electromagnetic tracking device for the arm and hand segment or a CyberGlove for hand kinematics. The "teacher" shows the patient the trajectory of the end-point (hand) path for the movement to be reproduced. Frequency of visual feedback, speed of motion, degree of movement synchronization and other aspects of the teacher-patient relationship can be modulated. Data from eight chronic post-stroke patients demonstrated variable improvements on clinical measures of upper extremity function including strength.

Piron et al. [34] used a virtual reality task to assess functional motor progress of a group of 20 post-stroke patients undergoing conventional rehabilitation. The patients were required to move an envelope instrumented with a magnetic receiver to a virtual mailbox slot. The participant was provided with a view of the trajectory of the corresponding virtual envelope as it moved. Patients improved on reach velocity and reach duration with the changes related to improvements on a clinical measure of upper extremity voluntary movement. The authors suggest that the reach trajectory characteristics also improved although limited data were presented. Several questions however remain. Primarily, would similar changes in movement trajectories be observed if the subject did not "see" a virtual mailbox? Moreover, in this paradigm, the trajectory to the mailbox is only one aspect of the functional task while an equally, if not more important task component is the orientation of the envelope once it reaches the mailbox slot. This emphasizes the need to adequately characterize and represent the functional task to be practiced within the VE.

The Rutgers ankle and hand systems, both incorporating the haptic sense, were developed as assessment and intervention tools although there are limited clinical data available at this time regarding efficacy [see [35,36]]. The two systems combine force feedback with a virtual environment that requires subjects to complete various tasks such as a virtual PegBoard task as well as reach-to-grasp (hand system) or piloting a virtual airplane through loops (ankle system). Preliminary data suggest that the systems may be useful to augment rehabilitation in patients in the chronic phase following stroke. A recent study using the hand system demonstrated transfer of skills acquired with the VR system to a functional clinical outcome measure as well as improvement on a variety of movement parameters with greatest benefit recorded in the least impaired patients [37].

#### Exercise and pain tolerance

Chuang et al [38] compared physiological responses of the cardiovascular and respiratory systems during incremental exercise testing with and without VR in healthy older adults. A mechanically braked bicycle was linked to a visual virtual scene projected on a flatscreen display. The rate of subject movement on the bicycle matched the environmental flow on the screen and included a 5 km straight or curved road bordered by grass, trees, seashore background and street lamps. No differences were observed on submaximal and peak exercise responses but the cycling with the VR scenario resulted in longer mean values for cycling duration, distance and energy consumption. It is possible that performing the exercises while immersed in a comfortable environment resulted in an increased degree of relative tolerance.

Positive outcomes of virtual reality as a distractive technique have also been reported for physiotherapy treatment sessions. Hoffman and colleagues [39] report decreased anxiety and reductions in self-report of pain from a single-pediatric patient undergoing post-operative physiotherapy. The child underwent single event multi-level surgery including femoral de-rotation osteotomy, quadriceps tendon translocation and release of the Achilles and hamstring tendons. Children experience high levels of post-operative pain association with physiotherapy treatments despite standardized pharmacological analgesia. Effective use of VR as a non-pharmacological analgesia for patients post-surgery may result in greater therapy gains.

#### Assessment

Although the majority of VR environments that have been developed for assessment to date focus on daily living skills such as meal preparation [40], spatial memory [8] and cognitive function [41], specific applications have been developed for assessment of upper and lower extremity motor function, balance and locomotion. For example, two separate assessment approaches using the PHANTOM haptic interface, a 6 degree of freedom measuring device for positional input that provides feedback force in translation and rotation have been developed. Broeren et al [42,43] used a relatively simple task requiring the user to reach for, grasp and move the visual representation of the device from a home position to nine separate locations in the visual field. Preliminary data suggest that this is a potential tool for identifying specific deficits of movement such as timing or accuracy that vary across patients. A more complex use of the technology, labyrinth navigation, has been used to isolate more subtle aspects of movement in patients with neurological disease including tremor amplitude and frequency, movement control, and speed of advancement through the labyrinth [44].

Assessments can be developed using VR technologies that will provide objective, repeatable and quantitative results. Standardized instructions, non-varying environmental cues, tasks and feedback can be achieved. In the extreme condition, interactions are limited to those between the patient and a virtual assessor. Since the devices are programmable, varying the complexity of assessment tasks is relatively trivial allowing for batteries of simple and more complex tasks to be developed. For example, an upper extremity assessment scale may include tasks requiring self-selected motion as well as responses to force perturbations permitting assessment of feedback limb control.

#### Access to rehabilitation

The degree of functional movement outcome achieved by therapy is often sub-optimal since intensive therapy is limited by resource allocation and access. For many individuals, such as traumatic brain injury survivors, access to therapy is terminated once a level of function is achieved even if residual deficits remain. For other individuals, even when therapy is available such as during in-patient neurological rehabilitation, low levels of interaction between the patient and environment have been reported [45,46]. For example, Tinson [46] reported that individuals post stroke typically spent only 20–60 minutes per day in formal therapy. Common problems influencing the degree of interaction include boredom, fatigue, lack of motivation and lack of cooperation in attending therapy [47]. Clinicians agree that such problems are undesirable and restrict progress in rehabilitation. Increasing interaction is seen as vital to effective rehabilitation, a fact borne out by experimental studies of recovery after brain damage [48]. Development and incorporation of virtual reality applications in rehabilitation may increase the possibility of stimulation and interaction with the world with potentially little or no increase on the demands of staff time. Virtual reality may provide interesting and engaging tasks that are more motivating than formal repetitive therapy. In fact, our recent experience comparing participant perceptions of exercise programs strongly suggest there is added benefit with VR compared to a conventional program (M Thornton et al, unpublished data). For example, the son of a TBI survivor participating in a VR balance retraining program noted *We have tried in the past to have him involved in things but he seemed uninterested. With these exercises *(referring to a VR-exercise balance retraining program) *he was trying to explain what he was doing, he was interested in what he was doing, he was looking forward to going*.

## Summary

An exponentially increasing number of distinct VR applications are being developed for intervention and assessment of a broad range of motor rehabilitation needs including upper and lower extremity function, balance and locomotion. Although the initial VR rehabilitation applications that were developed, in particular applications using video capture technologies and most HMDs, were subjected to relatively prohibitive entry level costs associated with the technology, recent developments in technology have made the number of low-cost multisensory VR applications increasingly available. Significant decreases in the costs associated with HMDs and motion trackers, desktop computers and certain haptic devices, are facilitating the development of low cost off-the-shelf applications.

The applications reviewed in this paper have demonstrated improvements of specific motor function with certain populations. It is clear that many of the applications that have been developed, for example gait trainers, will serve a specific rehabilitation niche. These devices have the potential to significantly extend our current understanding of movement and therapy and may substantially impact delivery of rehabilitation interventions. Critical for continued successful integration of virtual reality in motor rehabilitation is the need for the ongoing development and use of the technology to be based on clear understanding of the complexity of voluntary movement [49]. Sensorimotor integration, movement production, learning and transfer as well as psychosocial benefits are critical issues to address in ongoing and future studies. Of crucial importance is the fundamental question "Can the same objective be accomplished with a simpler approach". Prior to adoption of novel rehabilitation approaches including virtual reality based applications, users must assess whether the VR technology will provide any additional benefits to that of well trained and experienced therapists.
